# Evaluation of different surface treated implants after provisionalization: A 6-month prospective study

**DOI:** 10.34172/japid.2023.019

**Published:** 2023-11-06

**Authors:** Anshdha Shah, Amitabh Srivastava, Shivam Yadav, Charu Tandon

**Affiliations:** ^1^Department of Periodontology, Sardar Patel Post Graduate Institute of Dental & Medical Sciences, Uttar Pradesh, India; ^2^Department of Dentistry, Autonomous State Medical College, Hardoi, India

**Keywords:** Immediate non-functional loading, Implant stability, Implant surface treatment, Osseointegration

## Abstract

**Background.:**

Replacing missing teeth with dental implants has become the best treatment option; therefore, clinicians need to understand the predictability of the treatment. Surface treatment of implants is one of the methods to improve osseointegration, thus improving the quality of treatment. Increasing esthetic awareness among patients has led to the popularity of immediate provisionalization of dental implants. This study investigated the effect of surface treatment on implant stability when loaded with immediate non-functional temporary prostheses and compared the superiority of one surface treatment over the other in terms of osseointegration by evaluating implant stability quotient (ISQ).

**Methods.:**

Twenty implants with different surface treatments were placed, i.e., resorbable blast media (RBM) surface and alumina blasted/acid-etched (AB/AE) surfaces. All the implants were non-functionally loaded, and ISQ was measured immediately after implant placement and 6 and 12 weeks after non-functional loading. Crestal bone levels, mPI, mSBI, and peri-implant probing depths were compared for both groups at 1, 3, and 6 months.

**Results.:**

At 12 weeks, all the implants showed desirable ISQ, indicating successful osseointegration. The increase in ISQ at 12 weeks was significantly higher for RBM implants compared to baseline, indicating a more predictable course of osseointegration. Crestal bone levels recorded at 1, 3, and 6 months did not significantly differ between the groups. All other parameters showed comparable values for both groups at all intervals.

**Conclusion.:**

Replacing missing teeth with dental implants with immediate non-functional restorations is a predictable treatment option.

## Introduction

 Implants are considered a suitable treatment modality to restore missing teeth, primarily focusing on esthetics in the anterior region. The conventional loading protocol incorporates a load-free healing period of four months, allowing for osseointegration to occur in an undisturbed environment.^1^ Immediate non-functional restoration of the implant shortens the treatment time with better patient acceptability as the prosthesis is delivered within two days of implant insertion with no direct occlusal load, minimizing the risk of excessive forces on the implant.^2^

 Many authors have identified implant primary stability as one of the most crucial clinical factors influencing the success of immediate loading.^3,4^ It has been defined as a sufficiently strong initial bone–implant fixation and is determined by the available bone and implant shape and size.^2^ Secondary stability is responsible for stability in the months after implant placement and is determined by implant surface characteristics and bone quantity and quality.^5^

 Various methods are used to evaluate the implant stability, including the percussion test, reverse torque test, and quantitative ultrasound. There are two available instruments by which the clinical stability of an implant can be estimated: the Periotest (PTV) and the Osstell^®^ device. The Osstell^®^ device measures the implant stability by resonance frequency analysis (RFA). RFA is a non-invasive method that measures implant stability as a function of bone–implant interface stiffness and can be used at various time intervals during the treatment.^6^

 Surface roughness (of treated implant surface) creates a roughened micro and nanostructure topography, leading to optimal cell adhesion and proliferation, improving osseointegration. It can be achieved by various methods, including acid etching, sandblasting with alumina, titanium oxide, silica, or resorbable biocompatible bioceramics (resorbable blasting media [RBM]).^3,4^ Coatings over the implants are generally not preferred as these coatings dissolve in vivo.

 ADIN Touareg-S^TM^ implant has an alumina oxide-blasted/acid-etched (AB/AE) surface treatment, which creates a roughened topography, and ADIN Touareg-OS^TM^ implant has a RBM surface treatment where the implant surface uses calcium phosphate bioceramics, which is highly biocompatible, enriching the implant surface with calcium, phosphorus, and oxygen.

 This study compared different surface-treated implants (RBM and AB/AE surfaces) with immediate non-functional restoration and showed the superiority of one surface over the other in terms of primary and secondary implant stability.

## Methods

###  Study design

 A prospective randomized clinical trial was conducted on 12 patients (9 males and 3 females) aged 20‒55. The patients with missing teeth in the anterior maxilla who desired replacement by dental implant were selected for the study. Informed written consent was obtained from all the subjects participating in the study. Approval was granted by the institutional ethics committee (731718/PERIO/IEC/03).

###  Inclusion criteria

1. Patients with missing teeth in the maxillary anterior region
2. Patients where adequate bone was available for implant placement of appropriate size

###  Exclusion criteria

1. Patients with anterior deep bite, tongue thrust, and bruxism to prevent excessive forces on the provisional restoration
2. Current smokers
3. Patients with increased crown height space
4. Cases where bone augmentation was required
5. Patients with immunocompromised conditions or any systemic condition that might interfere with healing

The implant sites in all the patients were randomly divided into two groups:

 Group A: 10 implant sites received RBM surface-treated (ADIN: Touareg-OS^TM^) dental implants

 Group B: 10 implant sites received AB/AE surface-treated (ADIN: Touareg-S^TM^) dental implants

 Implant sizes ( ≥ 3.5 mm, ≤ 10 mm) suitable for immediate restoration were used. Short dental implants or narrow platform implants were not used in the study.

###  Pre-surgical preparation

 In all the subjects, hard tissues were evaluated using radiographs (IOPAR/OPG/CBCT), and the appropriate implant size was determined. Diagnostic casts were made, and complete blood counts, bleeding and clotting times, and vital signs were evaluated for all the patients before surgery.

###  Surgical protocol

**Figure 1:**
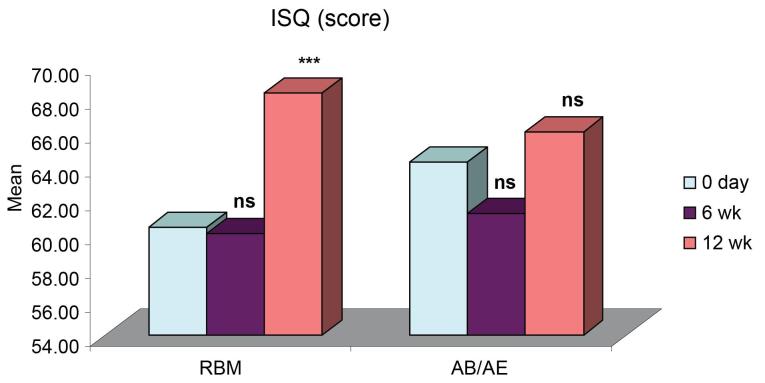
Intragroup comparisons of differences in mean ISQ

**Figure 2:**
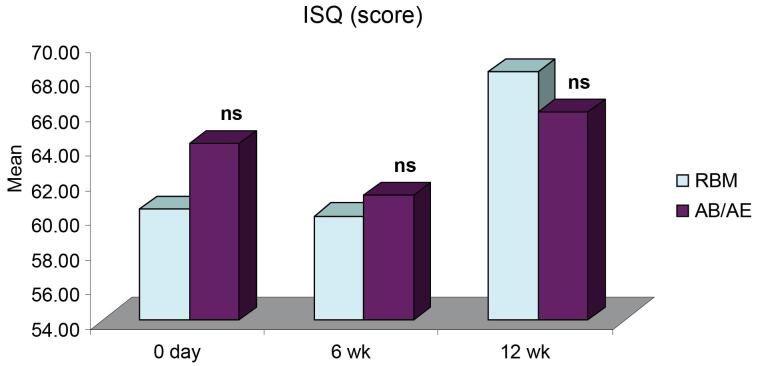
Intergroup comparisons of differences in mean ISQ score at different intervals

 A strict aseptic surgical protocol was followed for the surgery. The implant surgery was carried out conventionally, and an insertion torque in the range of 20‒30 N/cm was achieved for all the implants.^7^ Implant stability quotient (ISQ)was measured at the time of implant placement with the Osstell^®^ device. Provisionalization was carried out only when the ISQ was 55‒60(Figure 1).^8^ The regular platform abutment was secured to the implant. Then, sutures (3-0 silk) were placed adjacent to the abutment to ensure primary closure of the soft tissue surrounding the abutment (Figure 2). The patients were given postoperative instructions. Sutures were removed after seven days.

###  Provisionalization

**Figure 3:**
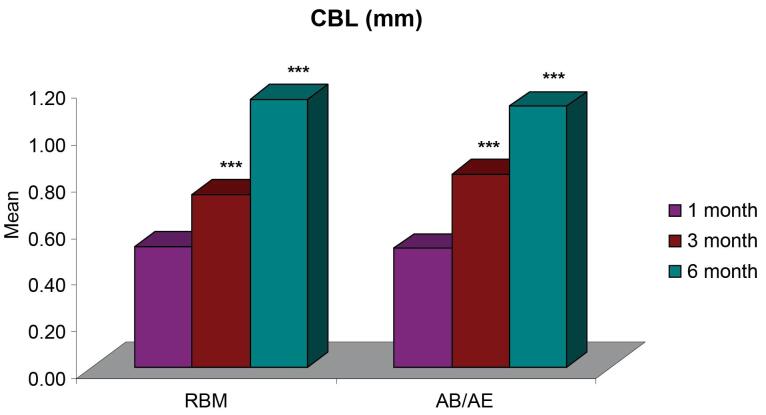
Intragroup comparisons of differences in mean CBL (mm) at different time intervals

 The impression for the temporary crown was taken on the same day with a rubber-based (Aquasil, Dentsply) and light body impression material (3M, ESPE). An acrylic prosthesis was fabricated, which was polished and reduced palatally such that it did not contact the teeth from the opposing arch at any point (Figure 3).

 The patients were recalled the next day; the acrylic prosthesis was cemented using temporary cement (non-eugenol based). The patients were instructed not to bite or chew using the anterior teeth with the temporary restoration to facilitate non-functional loading. The patients were recalled after 4, 6, and 12 weeks.

###  ISQ measurements

**Figure 4:**
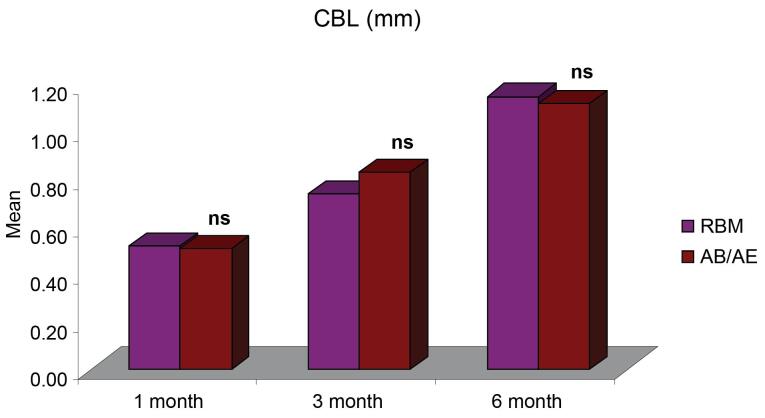
Intergroup comparisons of differences in mean CBL (mm) at different intervals

**Figure 5:**
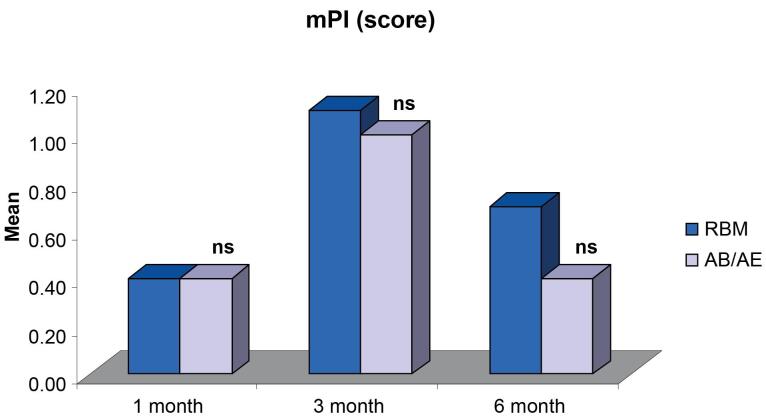
Comparisons of differences in mean mPI scores between the groups at different intervals

 ISQ was recorded using the Osstell^®^ device consisting of a peg, transducer, and monitor. To obtain the ISQ, the peg was screwed onto the implant, the transducer was held 1‒2 mm away from the smart peg, and the value was recorded in three directions: apically, mesially, and distally. The greatest value was considered. The values were obtained on a scale of 0–100 (Figures 4 and 5).^9^ The ISQ was measured at the time of surgery and 6 and 12 weeks after implant placement.^10,11^

###  Crestal bone evaluation

 Crestal bone levels were measured by IOPAR and recorded with a parallel cone technique using a photostimulable phosphor plate (PSP). Measurements were made from a predefined reference point, i.e., the implant shoulder to the first implant‒bone contact on the mesial and distal surfaces of the implants, and a mean value per implant was calculated.^12^ The distance between the implant shoulder and the most coronal implant–bone contact point was measured to the nearest 0.1 mm using VistaScan Mini Plus^TM^ (Durr- Dental, Germany) on a #2 image plate. Scores were recorded 1, 3, and 6 months after implant placement.

###  Prosthesis fabrication

 The final restoration was fabricated at 12 weeks if the ISQ value was between 65‒70. The abutment was removed, and a transfer coping was screwed onto the implant. Closed tray impression was recorded using putty/wash rubber base impression material (Aquasil, Dentsply). The abutment was placed again, and a closed tray transfer coping-implant analog assembly was seated in the impression.

**Figure 6:**
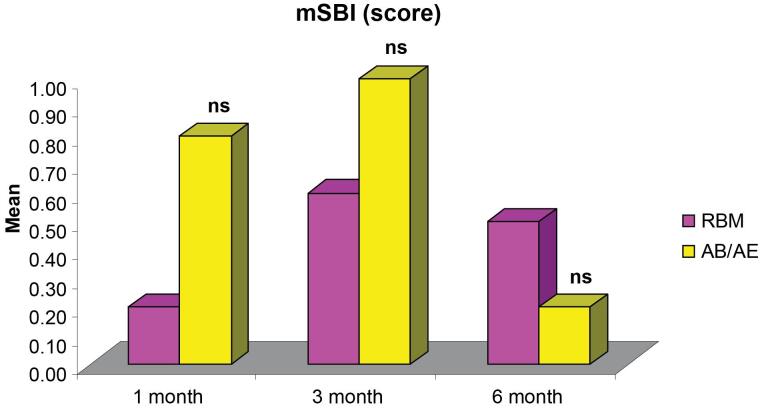
Comparisons of differences in mean mSBI scores between the groups at different intervals

 The laboratory steps were followed to fabricate a porcelain fused-to-metal crown. The crown was then glazed, polished, and delivered to the patient (Figure 6).

###  Parameters assessed

 ISQ was recorded on day 0 and 6 and 12 weeks after implant placement using the Osstell^®^ device on a scale of 1‒100. The crestal bone levels were evaluated at 1, 3, and 6 months postoperatively. Modified plaque index (mPI)^12^ and modified sulcus bleeding index (mSBI)^13^ were measured 1, 3, and 6 months after implant placement. The peri-implant probing depth was recorded on four surfaces, and the mean was calculated. The readings were made at 1, 3, and 6 months.^14^

 Data were summarized as mean ± SE (standard error of the mean). The groups were compared by independent Student’s t-test and ANOVA. The significance of the mean difference within (intra) and between (inter) the groups was analyzed by post hoc Tukey tests after ascertaining normality by Shapiro-Wilk’s test and homogeneity of variance between groups by Levene’s test. The groups were compared by chi-squared test. A two-tailed (α = 2) *P* < 0.05 was considered statistically significant. Analyses were performed using SPSS.

## Results

 All the implants placed were non-mobile and functional until the completion of the study. None of the implants showed peri-implant radiolucency or signs of peri-implantitis.

 ISQ for both groups was comparable at all intervals. It showed a slight decrease six weeks after implant placement and an increase by the 12th week. The mean ISQ for the RBM group was 60.40 ± 2.00 (day 0), 60.00 ± 1.98 (6 weeks), and a significant increase to 68.30 ± 1.29 at 12 weeks. The mean for the AB/AE group was 64 ± 1.74, which decreased to 61.20 ± 1.82 at six weeks and then increased to 66 ± 1.22 by the 12th week (Table 1). All the implants exhibited a stable ISQ (equivalent to or higher than the value obtained at implant placement) at 12 weeks. There was no significant difference in the ISQ values for the RBM and AB/AE groups at any of the intervals (Figure 1). However, group A (RBM) implants showed a significant increase in ISQ values at 12 weeks compared to both baseline (day 0) and six weeks. In contrast, the increase in ISQ values in group B (AB/AE) implants was only slightly significant at 12 weeks compared to six weeks (Figure 2), and the increase from baseline was not significant.

**Table 1:** ISQ scores (Mean ± SE) of the two groups over the periods

**Period**	**RBM (n=10)**	**AB/AE (n=10)**
0 day	60.40 ± 2.00	64.20 ± 1.74
6 wk	60.00 ± 1.98	61.20 ± 1.82
12 wk	68.30 ± 1.29	66.00 ± 1.22

 The mean crestal bone loss (CBL) measured from the shoulder of the implant for group A was 0.52 ± 0.04 mm one month after implant placement, 0.74 ± 0.04 mm at three months, and 1.15 ± 0.06 six months after implant placement. For group B, the mean CBL was 0.51 ± 0.03 mm one month after implant placement, 0.83 ± 0.04 mm at three months, and 1.12 ± 0.05 mm six months after implant placement. The CBL around implants for both groups was < 1.2 mm at six months (Figure 3, Table 2), and it did not show any significant difference between the two groups 1, 3, and 6 months after provisionalization and final prosthesis (Figure 4).

**Table 2:** CBL, mPI score, mSBI score, and PIPD at different time intervals

**Parameter**	**RBM Group (n=10)** **(Mean±SE)**			**AB/AE Group (n=10)** **(Mean±SE)**		
	**1 month**	**3 months**	**6 months**	**1month**	**3 months**	**6 months**
CBL (mm)	0.52 ± 0.04	0.74 ± 0.04	1.15 ± 0.06	0.51 ± 0.03	0.83 ± 0.04	1.12 ± 0.05
mPI score	0.40 ± 0.16	1.10 ± 0.18	0.70 ± 0.15	0.40 ± 0.22	1.00 ± 0.15	0.40 ± 0.16
mSBI score	0.20 ± 0.13	0.60 ± 0.16	0.50 ± 0.17	0.80 ± 0.20	1.00 ± 0.15	0.20 ± 0.13
PIPD (mm)	1.90 ± 0.10	2.10 ± 0.10	2.00 ± 0.21	2.10 ± 0.10	2.20 ± 0.13	2.30 ± 0.21

 The comparison of mean mPI demonstrated no significant differences between groups 1, 3, and 6 months after provisionalization and final prosthesis (Figure 5). The mean mPI at six months (0.70 ± 0.15 for the RBM group and 0.40 ± 0.16 for the AB/AE group) (Table 2) did not indicate the presence of any factors that may cause disease.

 The comparison of mean mSBI demonstrated no significant differences between the groups at all intervals (Figure 6). The mean mSBI at six months (0.50 ± 0.17 for the RBM group and 0.20 ± 0.13 for the AB/AE group) (Table 2) indicated the presence of healthy peri-implant soft tissues.

**Figure 7:**
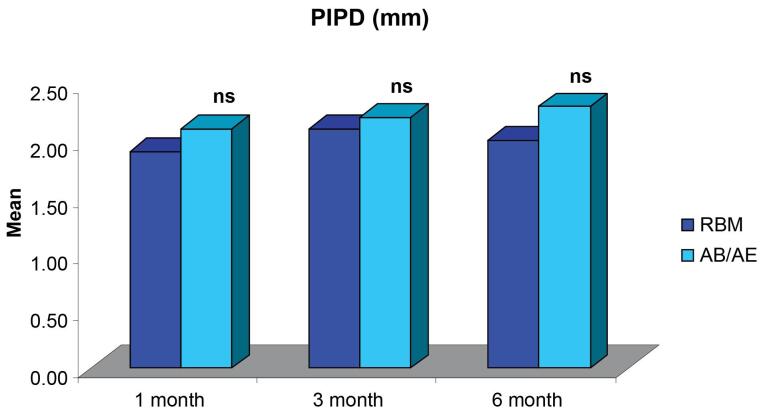
Comparisons of differences in mean PIPD (mm) scores between the groups at different intervals. ***P < 0.001 – highly significant; **P < 0,001 – moderately significant; *P < 0.01 – significant; ^ns^*P* > 0.05 – not significant

 The comparison of mean Peri-implant Probing Depth (PIPD) demonstrated no significant differences between the groups at all intervals and was within the limits for a successful implant (Figure 7).

## Discussion

 Conventional implantology follows an approach where the implant undergoes a load-free healing period of several months, allowing it to osseointegrate without any forces or micromovements on the implant. This poses a problem in the esthetic zone, where the patient desires a fixed interim prosthesis. The desire to fulfill the esthetic need led to the introduction of the concept of immediate loading, defined as applying a load using an occluding or a non-occluding restoration within 48 hours of implant placement. Implant loading with a non-occluding restoration is known as immediate non-occlusal loading, and it is loaded using lip and tongue pressure and contact with food but not from the occlusal forces of the opposing dentition.^2^

 The immediate restoration helps avoid a second-stage surgery and the application of forces that allow adequate loading ( < 150 µm) leads to adaptive remodeling of bone around the implant.^15^ Controlled mechanical loads lead to increased osteoblast and osteocyte production, leading to accelerated formation of spongy bone. There is also enhanced production of type I and type III collagen, and there is a preferential alignment of collagen fibers, which helps determine the quality of bone.^16^

 Melsen and Lang^17^ reported significantly higher bone apposition around loaded implants than the unloaded implants. Vandamme et al^16^ reported that significantly more osteoid was found in contact with the implant with loaded conditions compared to no loading.

 The prerequisite for immediate loading is primary stability. The primary stability, as determined by the radiofrequency analysis, is determined by the bone‒implant contact, and the ISQ value helps determine whether a prosthesis can be delivered immediately or not. The factors that determine the primary stability are the implant design, implant thread design, and bone density.^18^

 Threaded, conical implants^19^ with variable thread designs^20^ have shown favorable results. In this study, the implant used (Adin Touareg^TM^) is a tapered threaded implant with variable threads (square and spiral), with a 2-mm depth, allowing self-cutting and better load distribution.

 As the healing process continues, the primary stability is replaced by secondary stability, which determines the implant’s stability after months and years of placement and is influenced greatly by the surface characteristics of the implant. Compared to smooth surfaces, textured implant surfaces exhibit more surface area for integrating with bone. Experimental studies have demonstrated a faster rate and higher degree of bone formation for rougher implants than for implants with machined surfaces.^21^

 The ISQ value attained during surgery begins to decrease as the healing process starts with an inflammatory response. ISQ continues to decrease, attaining the lowest value at nearly three weeks, corresponding with the formation of a fibrocartilaginous network (osteoid) around the implant. This pattern lasts 4‒6 weeks or until the woven bone formation lasts. At around 12 weeks, as the spongy bone is formed, an ISQ value equivalent to the ISQ at the time of implant placement is achieved.^22,23^ Ersanli et al^23^ reported a similar trend of decrease in ISQ readings six weeks after surgery and a recovery of ISQ levels identical to day 0 at the time of loading.

 The increase in ISQ at 12 weeks from baseline was higher for group A (RBM) than for group B (AB/AE). This significantly higher increase in group A (RBM) can be attributed to the biocompatible calcium and phosphorous particles on the RBM surface, consistent with a study by Witek et al^24^ in a dog model, where the authors reported removal torque values were nonsignificant for both the groups at 1- and 3-week intervals, but at the 6th week, the values became significantly higher for RBM surface.

 CBL is influenced by numerous variables related to surgical trauma, prosthetic considerations, implant designs, bone substratum, patient habits, and implant‒abutment connection. The CBL pattern was similar for both RBM and AB/AE groups without any significant difference, indicating that surface treatments did not significantly affect the hard tissue changes. The findings of the present study are consistent with a study by Vandeweghe et al,^25^ where the authors observed a mean CBL of 1.19 mm at six months and concluded that bone remodeling continued for six months, after which no further changes were observed in the crestal bone levels.

 As soon as a restorative surface is introduced into the oral cavity, it is susceptible to plaque formation. Since, in this study, the implant received immediate restoration, it was exposed to plaque immediately after placement. Plaque is considered an important etiologic factor in the development of peri-implant mucositis, where the inflammation is confined to the soft tissues around a dental implant. The mean difference in mPI between both groups was 0.00 at one month, 0.10 at three months, and 0.30 at six months, with no significant difference between the two groups at any of the intervals. A study by Al-Dharrab^26^ showed a similar trend of increase in the mPI score, i.e., a score of > 2 at three months and then a decrease in the score at 12 months, i.e., a score of ≥ 1 but < 2 for implant-supported overdentures. The increase in the mPI values seen within the groups at 1 and 3 months might be attributed to the relatively rough surface of the temporary crown compared to the more polished surface of a porcelain fused-to-metal permanent restoration.

 The mSBI score increased from the first month to the third month in both groups, but the increase was not significant, and the values remained ≤ 1. The values decreased at six months compared to three months in both groups. Similar values were observed by Han et al^27^ with bleeding on probing on 0‒2 surfaces around the implant at 6-, 8-, and 12-week follow-ups.

 The PIPD within the groups did not differ significantly at any of the intervals for either of the groups (groups A and B), with no significant difference in PIPD between the groups at any of the intervals. The average values remained < 3 mm at all time intervals. Similar findings were seen in a study by Han et al,^27^ where the probing depth around implants ranged between 1 and 3 mm at 6, 8, and 12 weeks. Sekar et al,^28^ reported a similar increase in probing depth from baseline to six months. The probable reason for the increase in the PIPD seen in our study could be undetected subgingival plaque accumulation. Another possible explanation for increased PIPD could be the remodeling of peri-implant soft tissue to maintain “biological width,” as stated by Koutouzis et al.^29^

 Variations of observations in various studies may arise due to difficulty in oral hygiene maintenance after prosthetic loading, leading to inflammation and bleeding on probing. Variations in the finish of prosthetic components, patient awareness, plaque control measures, and compliance with oral hygiene reinforcement instructions also play a role.

 The study’s outcomes provided the necessary evidence to believe that the immediate provisionalization in the maxillary esthetic zone is a safe and feasible treatment option, as no failures were seen during the six-month follow-up period of the study. The roughened implant surface helps achieve good primary stability and could be considered suitable to bear the forces exerted by the soft tissues of the oral cavity and the forces encountered during swallowing. The stability after six months of function in both implant types used in the present study was comparable. However, calcium and phosphorous particles on the RBM surface positively affected osseointegration, leading to increased implant stability, as seen in the study.

## Conclusion

 Within the limitations of the present study, it can be concluded that dental implants with immediate non-functional restorations in the maxillary esthetic zone are a predictable option for replacing missing teeth with the added advantage of meeting the esthetic needs of the patient. When compared, RBM and AB/AE implants showed good clinical performance when provisionalized immediately and subjected to mild mechanical forces. RBM surfaces provided slightly better implant stability than alumina-blasted/acid-etched surfaces. Further research is necessary with larger sample sizes, longer follow-up periods, and more clinical and radiographic parameters to validate the results of this study.

## Acknowledgments

 We would like to thank the Department of Periodontology and the Department of Prosthodontics, SPPGIDMS, for supporting this study.

## Competing Interests

 The authors declare that they have no financial and non-financial competing interests concerning the publication of their work during submission.

## Consent for Publication

 The consent was obtained from all the subjects.

## Data Availability Statement

 The datasets used and/or analyzed during the current study are available from the corresponding author upon reasonable request.

## Ethical Approval

 Approval was acquired from the institutional ethical committee with approval number 731718/PERIO/IEC/03.

## Funding

 This research received no specific grants from funding agencies in the public, commercial, or not-for-profit sectors.
